# Diagnostic accuracy and safety of ultrasound-guided percutaneous core needle biopsy among children with extra cranial solid masses at a tertiary care hospital in Karachi, Pakistan

**DOI:** 10.12669/pjms.41.1.9777

**Published:** 2025-01

**Authors:** Fatima Ambreen Imran, Abdul Razaque, Aisha Mehwish Qureshi, Muhammad Rafie Raza

**Affiliations:** 1 Fatima Ambreen Imran, FCPS Department of Pediatric Oncology, Indus Hospital and Health Network, Karachi, Pakistan; 2 Abdul Razaque, FCPS Department of Radiology, Indus Hospital and Health Network, Karachi, Pakistan; 3 Aisha Mehwish Qureshi, MBBS Department of Pediatric Oncology, Indus Hospital and Health Network, Karachi, Pakistan; 4 Muhammad Rafie Raza, FCPS Department of Pediatric Oncology, Indus Hospital and Health Network, Karachi, Pakistan

**Keywords:** Ultrasound-guided core needle biopsy, Extra cranial solid masses, Diagnostic biopsy, Diagnostic accuracy, Procedural safety, Pediatric population

## Abstract

**Objective:**

To determine the diagnostic accuracy and procedure safety of ultrasound-guided core needle biopsy of extra cranial solid masses in the pediatric population.

**Method:**

A cross-sectional survey was conducted by the Department of Pediatric Hematology /Oncology and Radiology at Indus Hospital and Health Network Karachi from August 2022 to April 2023. A total of 118 pediatric patients, from age one month to 18 years, with extra cranial solid masses were studied. Data was analyzed using SPSS version 24.

**Results:**

The median age of study participants was six years with IQR = 7.2 years. Among all study participants 62.7% (n=74) were male while 37.3% (44) were female. Out of all biopsies 94.07% (n=111) were conclusive and provided an accurate diagnosis of the tumor while only 5.63% (n=07) biopsies remained inconclusive. No serious post-procedure complication was reported. Pain on the site of biopsy was the most post-procedure complain reported by 34.7% (n=41) of all the study participants. Forty-two percent of the study participants never reported any post-procedure complaints after ultrasound-guided biopsy. The diagnostic accuracy was significantly higher for malignant lesions as compared to non-malignant lesions.

**Conclusion:**

Ultrasound-guided percutaneous core needle biopsy had high diagnostic accuracy and a good safety profile among children with extra cranial solid masses in private tertiary care hospital in Karachi Pakistan.

## INTRODUCTION

An estimated 19.3 million new cases of cancer are diagnosed each year on a global level^1^ out of which 400,000 cases are reported in children less than 15 years of age.^2^ Of all the malignancies, the leukemias/lymphomas (~35%) are the most frequent followed by CNS malignancies in approximately 20% of the cases and then the solid tumors (~15%-blastomas and ~10%-sarcomas).^3^

The diagnosis and treatment of tumors require the histopathological examination of the biopsy sample to reach an accurate diagnosis and guide tumor management.^4^ Open tumor biopsy is widely used as a traditional approach for tumor biopsy of extra cranial solid masses among children.^5^ However, the high risk of complications and related morbidity is a major challenge associated with this conventional technique.^5^,^6^

The complications of surgical biopsy or open tumor biopsy may range from postoperative infection or sepsis to hospital readmission and even death.^6^ With the advancement in imaging sciences; core needle biopsy has emerged as a safer, quicker, and less invasive alternative to surgical/excisional biopsy of tumors and has been reported as a safer method among pediatric population.^7^,^8^ However, the use of ultrasound (US)- guided core needle biopsy for investigation of solid masses in the pediatric population requires local evidence regarding its diagnostic accuracy as well as safety in the local context.^4^ Literature suggests that core needle biopsy is a relatively safer alternative and avoids many post-procedure complications however, it can compromise the diagnostic yield and accuracy of the biopsy hence can compromise the patients’ diagnosis and disease management.^9^-^11^

Nevertheless, there is a severe dearth of evidence regarding the diagnostic accuracy and safety of core needle biopsy in the pediatric population of Pakistan. Therefore, this study was conducted at Indus Hospital & Health Network (IHHN), Karachi among pediatric patients presenting with extra cranial solid masses. This study determined the diagnostic accuracy and procedure safety of US-guided core needle biopsy for all pediatric extra cranial solid masses, from in-patient and out-patient departments.

## METHODS

A cross-sectional study was conducted by the Departments of Pediatric Hematology/Oncology and Radiology at IHHN from August 2022 to April 2023. IHHN is a tertiary care not for profit hospital offering diagnostics and treatment services to more than 1200 new cases registered every year in Pediatric hematology/Oncology department. Before enrolment in the study all possible risks and benefits were explained to parents in detail at the time of obtaining informed consent. Voluntary informed consent was taken from parents/guardians (if the patient was younger than 10 years) of patients and assent from patients (>10 years-<18 years).

### Ethical Approval:

It was obtained from the Institutional Review Board of Indus Hospital, Karachi (Ref# IHHN_IRB_2022_04_005, Dated: August 16, 2022).

The sample size for post-procedure complications was also calculated at a 5% level of significance using OpenEpi software with an anticipated proportion of 4.1% for post-procedure complications and a minimum sample size of 118 was obtained.^12^ Pediatric patients aged one month to 18 years of age with extra cranial solid masses and not fit for primary oncology surgery or pediatric patients with localized or metastatic disease presenting directly or being referred by pediatricians or oncologists for core needle biopsy for whom the origin of the tumor was unclear and required further histological verification were included in the study on a non-probability convenient basis.

Other factors that played a role in the selection of the patients included age (>1 month), palpable soft tissue mass or nodes which were visible on ultrasound, easily accessible lesion, size at least 1-2 cm in diameter to accommodate the biopsy needle, and hemodynamically stable patient. Most of the patients were suspected with malignant disease on Computed tomography (CT scan) or Magnetic resonance imaging (MRI) with contrast. Any patient otherwise meeting inclusion criteria but found unfit for optimal core needle biopsy due to the inaccessible location of the tumor as identified on imaging, having any bleeding disorders, was excluded from the study.

The radiology department has all basic infrastructure resources including dedicated procedure room, sterile equipment, patient monitoring devices. Personnel involved in the team included experienced interventional radiologist, medical assistant, anesthesiologist, and a doctor in the daycare for post-procedural recovery monitoring till discharge for out-patients. A mandatory pre-procedure screening was conducted to assess the medical eligibility of the participant for the US guided core needle biopsy, including hemoglobin, platelet count, coagulation profile and chest x-ray. Before performing the biopsy, each lesion was examined using conventional and Doppler ultrasound (U/S Philips model # Epiq Elite) to determine the best sampling site. Colored Doppler images were used to identify major blood vessels. Biopsies were performed with 18 gauge semi-automatic trucut biopsy needle. Minimum three and maximum five tissue cores were taken during the procedure.

The samples were sent for histopathology and immunohistochemistry. Every patient was kept under observation with regular monitoring of the vital signs and immediate complications (fever, pain, swelling, redness, tenderness, nausea/ vomiting, bleeding, bleeding requiring transfusion) for at least two hours after the procedure and then discharged to home or shifted back to the respective unit (in case of in-patient) with necessary instructions. The outpatients were then instructed to follow-up after a week to evaluate for the presence of late complications (swelling, pain etc.) which were then managed accordingly.

### Data Collection:

Data was collected using structured proforma including variables such as age, gender, health-related characteristics including indication for biopsy, any prior health issues, site of lesion, and type of patient (in-patient versus out-patient). Data regarding diagnostic outcome, histological findings, and type of post-procedure complication were collected seven days after the procedure in the consulting clinic and directly from admitted patients. The patients who could not be followed after one week were contacted telephonically and inquired about any possible complications and asked to come for staging work up and treatment.

### Data Analysis:

Data was analyzed using SPSS version 24.0. The distribution of the data was determined using the Shapiro-Wilk test. Descriptive statistics were calculated for socio-demographic characteristics, health-related characteristics, and procedure-related complications. The Chi-square test was applied to assess the possible association between post-procedure complications and socio-demographic and health-related characteristics. Non-diagnostic results included situations such as insufficient cellularity, necrosis, and blood or tissue artifacts.

The diagnostic yield was calculated as the proportion of diagnostic biopsies among the total number of biopsies performed. The diagnostic accuracy of ultrasound-guided core needle biopsy was calculated as the proportion of biopsies resulting in accurate diagnosis among those being conducted. Comparisons between diagnostic accuracies in diagnosing malignant and non-malignant lesions were also conducted using a Chi-square test of significance. A p-value of 0.05 or less was considered statistically significant.

## RESULTS

In total, 118 pediatric patients presenting with extra cranial masses were included in the study. The median age of study participants was six years (IQR: 4.0-11.3 years). Of the total,62.7% (n=74) of all study participants were male while 37.3% (44) were female. More than 50% of study participants were in-patients as they were admitted to the hospital at the time of recruitment in the study. The most common site or location of biopsy was the abdomen accounting for 49.2% (n=58) of all tumors, followed by head and neck tumors contributing to 23.7% (n=28). Approximately, 99.2% (n=117) of included tumors were primary in origin and only 0.8% (n=1) were metastatic lesions. All study participants received some form of anesthesia or sedation during US guided biopsy with 50% (n=59) of the participants were given only sedating drugs followed by 42.3% (n=50) of participants receiving general anesthesia (Table-I).

**Table-I:** Socio-demographic and health-related characteristics of the study participants (n=118).

Variables	Frequency, n (%)
Age, Median (IQR)	6.0 (4.0-11.3)
subgroups based on age (years)	
<5	57 (48.3)
5.1 - 10	30 (25.3)
10.1 - 16	31 (26.3)
Gender	
Male	74 (62.7)
Female	44 (37.3)
Type of Patient	
In-patient	63 (53.4)
Out-patient	55 (46.6)
Tumor Location	
Abdomen	58 (49.2)
Head and Neck masses	28 (23.7)
Pelvis	13 (11.0)
Chest	07 (5.9)
Limbs/ Muscles	04 (3.4)
Back/spine	01 (0.8)
Indication for Biopsy	
Primary Tumor	117 (99.2)
Metastasis	01 (0.8)
Anesthesia administered for biopsy	
Sedation	59 (50.0)
General Anesthesia	50 (42.4)
Local Anesthesia	09 (7.6)
Required hospital admission	
Yes	37 (31.4)
No	81 (68.6)
Remarkable findings*	24 (20.4)
No Remarkable findings	94 (79.6)
Prothrombin time	
Median (IQR)	11.2 (10.8-12.1)
APTT	
Median (IQR)	24.0 (22.0-27.1)
Platelets Count	
Median (IQR)	405.0 (268.5-565.3)

* Platelet counts were low in 2 patients, so their biopsy was done after platelet transfusion.

A total of 94.1% (n=111) of all biopsies conducted were found to be conclusive. Overall, 93.6% (n=104) of all the conclusive biopsies were reported as malignant disease while only 6.4% (n=07) were found to be non-malignant disease. Non-Hodgkin lymphoma, sarcomas (bone and soft tissue), neuroblastoma, Hodgkin lymphoma, and Wilm’s tumor were the most common diagnoses with a proportion of 32% (n= 33), 19.7% (n=19), 11.5%(n=12), 9.6% (n=10) and 7.7% (n=8) respectively. The repeat biopsy of seven inconclusive biopsies further resulted in conclusive outcomes for four more study participants i.e. they were diagnosed with synovial sarcoma, Wilms tumor (relapse), germ cell tumor, and Hodgkin’s lymphoma (relapse).

However, three participants` outcomes remained inconclusive due to inadequate sample and tissue necrosis. No serious post-procedural complaints were reported. Pain on site of biopsy was the most reported post-procedure complain reported by 34.7% (n=41) of subjects. About 42.4% (n=50) of the study participants did not report any post-procedure complication following the US guided core needle biopsy procedure (Table-II).

**Table-II:** Ultrasound-guided biopsy of extracranial tumors in pediatric patients-diagnostic outcomes and post-procedure complaints.

Variables	Frequency, n (%)
Biopsy Outcomes (n=118)	
Conclusive	111 (94.1)
Non-Conclusive	07 (5.9)
Frequency distribution for Malignant conclusive biopsies (n=104)	
Non-Hodgkin lymphoma	33 (32.0)
Neuroblastoma	12 (11.5)
Hodgkin lymphoma	10 (9.6)
Wilm`s tumor	08 (7.7)
Ewing sarcoma	08 (7.7)
Germ cell tumor	07 (6.7)
Spindle cell tumor/Synovial sarcoma	05 (4.8)
Rhabdomyosarcoma	04 (3.8)
Osteosarcoma	02 (1.9)
Ganglioneuroma	01 (0.9)
Myeloid Sarcoma	01 (0.9)
Others	07 (6.7)
Frequency distribution for Non-Malignant conclusive biopsies (n=7)	
Benign Reactive Lymph node	02 (28.5)
Chronic Osteomyelitis	01 (14.3)
Rosai-Dorfman Disease	01 (14.3)
Tyrosinemia (liver biopsy)	01 (14.3)
Lymphoproliferative disease	01 (14.3)
Fibromuscular Tissue	01 (14.3)
Post-procedure complaints (n=118)	
Pain	41 (34.7)
Fever	11 (9.3)
Pain and Fever	09 (7.6)
Pain and Swelling	05 (4.2)
Swelling	02 (1.7)
None	50 (42.5)
Bleeding	0
Bleeding requiring transfusion	0

**Fig.1:**
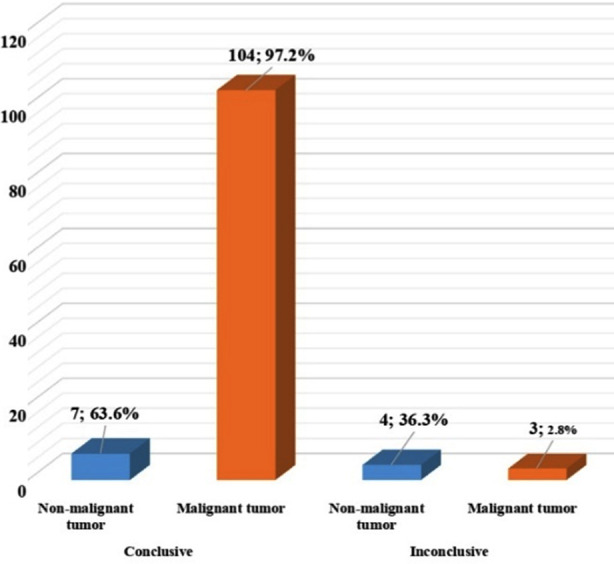
Comparison of diagnostic accuracy of ultrasound guided core needle biopsy for extra cranial masses in pediatric patients (n=118).

This study found statistically significant differences in the diagnostic accuracy of US-guided core needle biopsy for non-malignant versus malignant masses (p-value <0.01), shown in Fig.1. The diagnostic accuracy was comparatively high for malignant as compared to non-malignant masses. The association of the various demographic features with the presence of post-procedural complaints revealed no statistically significant results (Table-III).

**Table-III:** Association of parameters with the presence of post-procedural complications.

Parameter		Frequency	Post-procedural complains						p-value

Gender			None	Fever	Pain	Swelling	Fever, pain	Pain, swelling	
	Male, n (%)	44	34 (68.0)	7 (63.6)	21 (51.2)	1 (50.0)	7 (77.8)	4 (80.0)	0.477^£^
	Female, n (%)	74	16 (32.0)	4 (36.4)	20 (48.8)	1 (50.0)	2 (22.2)	1 (20.0)	
Age, Median(IQR)			5.0 (3.0-10.0)	4.0 (2.0-10.0)	7.0 (4.3-12.0)	3.5 (1.0-)	7.0 (2.0-13.0)	5.0 (3.5-12.0)	0.622^€^

£: Fisher-Exact test; €: Kruskal-Wallis test.

## DISCUSSION

This study was conducted to contribute to local evidence regarding the diagnostic accuracy and safety of US-guided core needle biopsy among children with extra cranial solid masses. The study found that US-guided core needle biopsy of extra cranial tumors among pediatric patients had considerably high diagnostic accuracy giving conclusive results for more than 90% of the study participants. The high diagnostic accuracy and high safety profile of US-guided biopsy in the pediatric population as reported by the current study provides worthy evidence supporting the valuable role of US-guided biopsy in the pediatric population with extra cranial tumors.^13^-^15^

The findings from this study are in line with the previous small-scale studies from Pakistan conducted in private hospitals of Karachi and Islamabad with a sample mix of adults and children and children below 12 years of age respectively.^14^,^16^ Both the mentioned studies reported a high diagnostic yield of US-guided biopsy with minimal complications. International data showed neuroblastoma, Wilm’s tumor, Non-Hodgkin’s; lymphoma, and germ cell tumors as the most common abdominal malignancies in pediatrics.^17^

Our current institutional practice does not include guided biopsies in case of renal tumors with typical radiological features as per the recent guidelines of UK CCLG Umbrella 2020. Thus Wilm’s tumor was not observed to be the second most common abdominal tumor in the present study. In addition, the findings of this study are well supported by international evidence coming from pediatric patients with a variety of extra cranial solid tumors Including; neuroblastoma, renal tumors, and liver and testicular tumors.^6^,^10^,^14^,^15^,^18^ The estimated diagnostic accuracy for diagnosing extra-cranial tumors in our study (97%) was observed to be quite similar to the evidence from similar populations (93.0% - 98.0%).^6^,^14^-^16^

In the current study among all the patients with extra cranial masses, only seven patients did not receive a conclusive pathological diagnosis on first US-guided core needle biopsy, while 104 patients obtained a conclusive diagnosis hence; showing a diagnostic accuracy of 97.1% for the malignant disease. The very high diagnostic accuracy for malignant disease in our sample is also comparable to previous evidence as reported by Loganathan and colleagues (93.6%) who assessed the diagnostic accuracy of US-guided biopsy among the pediatric population with solid extra cranial tumors.^14^ The comparable findings can be explained by sufficient sample size and inclusion of pediatric patients with a mix of different types of solid extra cranial tumors involving different body organs.

The study also found statistically significant differences in the diagnostic accuracy of US-guided core needle biopsy when compared between outcomes for malignant tumors versus non-malignant i.e. 97.1% vs. 61.5% respectively. This finding is in contrast to a previous study conducted by Wang H and colleagues who failed to find any association between the outcome of malignant and non-malignant disease.^15^ The unexpected relatively low accuracy for non-malignant diseases in our study can be explained by sampling errors due to the limited sample size and use of a non-probability convenient sampling technique. Hence, this adds to already available mixed evidence where diagnostic accuracy for non-malignant diseases may range from 87% to 100%.^6^ Large multi-center studies with larger sample sizes are required to further explore the potential utility of US-guided biopsy in case of non-malignant disease.

Similarly, this study could not find any significant differences in the post-procedure complications based on differences in socio-demographic characteristics. Our results showing the high safety profile of US-guided core needle biopsy to diagnose extra cranial masses among pediatric patients and the extremely low frequency of reported post-procedure complications provide evidence for possible standardization of this technique, where available for the pediatric population. The US-guided needle core biopsy can be done without the expense and availability of operation theatre and general anesthesia and helps avoid the need for surgical biopsy, providing an opportunity to avoid unnecessary morbidity among pediatric patients suffering from advanced tumors.

Moreover, replacing surgical biopsy with the mentioned procedure may reduce overall hospital stays as this procedure can be offered in daycare settings without any specific requirements for surgical facilities. An ultimate long-term consequence of reduction in cost, resources, and time as well as prevention of other potential hazards associated with hospital stay have been previously discussed and need to be further studied in an LMIC setting.^10^,^11^,^18^-^20^ Similarly the study found significant cost difference between US-guided core needle biopsy and surgical biopsy (approximately 5000 PKR vs. 70,000 PKR). This huge cost difference further validates the use of US-guided biopsy in comparison to surgical excision biopsy, particularly in a country which is riddled with financial constraints in every way possible. Specific costs may vary depending on location, institution, and patient factors. A systematic review also determined the safety of US-guided core needle biopsy for diagnosing head and neck tumors including the thyroid gland, salivary gland, and cervical lymph nodes as well as infectious lesions like extra pulmonary tuberculosis,^21^ as well as diagnostic tissue sampling for various anatomical areas of the head and neck.^21^

### Limitations:

This study had few methodological limitations. In this study, we only collected information from one particular private healthcare setting in Karachi through non-probability sampling to recruit the study participants with a disproportionate representation of pediatric patients from different age groups. Therefore, the findings from this study cannot be generalized until we explore this research question further by conducting large-scale multicenter studies with the representation of children from all age groups and application of probability or random sampling techniques with a sufficiently large sample.

Nevertheless, to the best of our knowledge this study is the first study in Pakistan presenting a considerably large sample of pediatric patients with tumors, their diagnostics outcomes from US-guided core needle biopsy, and post-procedure complications. Furthermore, the findings from this study can serve as a milestone to promote further research to establish generalizable concrete evidence regarding the potential utility of US-guided core needle biopsy as a gold standard of tumor diagnostics in the pediatric population.

## CONCLUSION

US-guided percutaneous core needle biopsy has high diagnostic accuracy and a promising safety profile among children presenting with solid extra cranial masses in a private tertiary care setting in Karachi, Pakistan. The diagnostic accuracy was significantly higher for malignant lesions as compared to non-malignant lesions.

### Author contributions:

**FAI:** Design of the study, acquisition analysis and interpretation of data, and drafted the article.

**AR:** Radiological assessment, and planned for guided core needle biopsy with safety and accuracy.

**AMQ:** Contributed to data acquisition. Literature search.

**MRR:** Critical Review, overall supervision.

All authors approve the final version and are accountable for the integrity of the study.

## References

[1] Ferlay J, Colombet M, Soerjomataram I, Parkin DM, Pineros M, Znaor A (2021). Cancer statistics for the year 2020: An overview. Int J Cancer.

[2] Bolous N, Mercredi P, Bonilla M, Friedrich P, Bhakta N, Metzger M (2024). Determining the cost and cost-effectiveness of childhood cancer treatment in Haiti. Ecancer.

[3] Ward ZJ, Yeh JM, Bhakta N, Frazier AL, Atun R (2019). Estimating the total incidence of global childhood cancer: a simulation-based analysis. Lancet Oncol.

[4] Garrett KM, Fuller CE, Santana VM, Shochat SJ, Hoffer FA (2005). Percutaneous biopsy of pediatric solid tumors. Cancer.

[5] Devin CL, Teeple EA, Linden AF, Gresh RC, Berman L (2021). The morbidity of open tumor biopsy for intraabdominal neoplasms in pediatric patients. Pediatr Surg Int.

[6] Chen Y, Huang Y, Feng J, Yang W, Wang H (2020). Reliability of ultrasound-guided percutaneous core needle biopsy in diagnostics of pediatric solid tumors. Authorea Preprints.

[7] Serati L, Morosi C, Barretta F, Collini P, Calareso G, Chiaravalli S (2021). Diagnostic yield and accuracy of image-guided percutaneous core needle biopsy of paediatric solid tumours: An experience from Italy. Pediatr Hematol Oncol J.

[8] Overman RE, Kartal TT, Cunningham AJ, Fialkowski EA, Naik-Mathuria BJ, Vasudevan SA (2020). Optimization of percutaneous biopsy for diagnosis and pretreatment risk assessment of neuroblastoma. Pediatr Blood Cancer.

[9] Metz T, Heider A, Vellody R, Jarboe MD, Gemmete JJ, Grove JJ (2016). Image-guided percutaneous core needle biopsy of soft-tissue masses in the pediatric population. Pediatr Radiol.

[10] Mohamed H, Pastor MC, Langlois S, Cowan KN (2022). Comparing safety and adequacy between surgical biopsy versus core needle biopsy in diagnosing neuroblastoma. J Pediatr Surg.

[11] Ilivitzki A, Sokolovski B, Assalia A, Benbarak A, Postovsky S, Glozman L (2021). Ultrasound-Guided Core Biopsy for Tissue Diagnosis in Pediatric Oncology: 16-Year Experience With 597 Biopsies. Am J Roentgenol.

[12] Ilivitzki A, Abugazala M, Arkovitz M, Benbarak A, Postovsky S, Arad-Cohen N (2014). Ultrasound-guided core biopsy as the primary tool for tissue diagnosis in pediatric oncology. J Pediatr Hematol Oncol.

[13] Faizan M, Anwar S, Khan S (2018). Demographics and Outome in Paediatric Non-Hodgkin Lymphoma: Single Centre Experience at The Children Hospital Lahore, Pakistan. J Coll Physicians Surg Pak.

[14] Loganathan AK, Jacob TJ, Matthew LG, Moses V, Keshava SKN, Priscilla AJ (2022). Efficacy of Core Needle Biopsy in the Diagnosis of Pediatric Extracranial Solid Malignancies: A 10-Year Study. J Indian Assoc Pediatr Surg.

[15] Wang H, Li F, Liu J, Zhang S (2014). Ultrasound-guided core needle biopsy in diagnosis of abdominal and pelvic neoplasm in pediatric patients. Pediatr Surg Int.

[16] Akhtar S, Riaz R, Waris R, Manzoor R (2018). Role of Ultrasound Guided Percutaneous Liver Biopsy Using Semi-Automatic Needle in Pediatric Liver Diseases. Pak Pediatr J.

[17] Yang H-B, Kim H-Y, Jung SE, Choi YH, Lee JW (2019). Pediatric minimally invasive surgery for malignant abdominal tumor: Single center experience. Medicine (Baltimore).

[18] Georgantzi K, Skoldenberg E, Janson ET, Jakobson A, Christofferson R (2019). Diagnostic ultrasound-guided cutting needle biopsies in neuroblastoma: A safe and efficient procedure. J Pediatr Surg.

[19] Short SS, Papillon S, Hunter CJ, Stanley P, Kerkar N, Wang L (2013). Percutaneous liver biopsy: pathologic diagnosis and complications in children. J Pediatr Gastroenterol Nutr.

[20] Minhas K, Roebuck DJ, Sebire N, Cho A, Patel PA (2023). Diagnostic yield and safety of ultrasound-guided percutaneous testicular biopsies in children. Pediatr Radiol.

[21] Kanodia KV, Vanikar AV, Nigam LK, Patel RD, Suthar KS, Gera DN (2015). Pediatric Renal Biopsies in India: A Single-Centre Experience of Six Years. Nephrourol Mon.

